# International stakeholder perspectives on One Health training and empowerment: a needs assessment for a One Health Workforce Academy

**DOI:** 10.1186/s42522-023-00083-4

**Published:** 2023-06-06

**Authors:** Ava Sullivan, Oladele Ogunseitan, Jonathan Epstein, Vipat Kuruchittham, Mabel Nangami, David Kabasa, William Bazeyo, Irene Naigaga, Olesya Kochkina, Winnie Bikaako, Nur Ahmad, Agnes Yawe, Christine Muhumuza, Rahmi Nuraini, Indira Wahyuni, Raja Adli, Saengduen Moonsom, Lai Huong, Phuc Pham, Terra Kelly, David Wolking, Woutrina Smith, One Health Workforce-Next Generation Consortium

**Affiliations:** 1 EcoHealth Alliance, New York, USA; 2 Department of Population Health and Disease Prevention, University of California Irvine, Irvine, USA; 3 Center for Innovation in Global Health, Stanford University, Stanford, USA; 4 Southeast Asia One Health University Network (SEAOHUN), Chiang Mai, Thailand; 5 Africa One Health University Network (AFROHUN), Kampala, Uganda; 6 Universiti Putra Malaysia, Serdang, Malaysia; 7 Indonesia One Health University Network (INDOHUN), Depok, West Java Indonesia; 8 Malaysia One Health University Network (MyOHUN), Serdang, Malaysia; 9 THOHUN-National Coordinating Office, Faculty of Tropical Medicine, Mahidol University, Bangkok, Thailand; 10 Vietnam One Health University Network (VOHUN); and Hanoi University of Public Health, Hanoi, Vietnam; 11 Vietnam One Health University Network (VOHUN), Hanoi, Vietnam; 12 One Health Institute, School of Veterinary Medicine, University of California, Davis, USA; 13 OHW-NG Consortium, c/o University of California, Davis, Davis, USA

**Keywords:** Assessment, Competency framework, Continuing professional development, Credential, Education, Employers, Employment, One Health, Stakeholders, Training, University networks, Workforce

## Abstract

**Background:**

One Health is defined as an integrated, unifying approach that aims to sustainably balance and optimize the health of people, animals and ecosystems; this approach attracts stakeholders from multiple sectors, academic disciplines, and professional practices. The diversity of expertise and interest groups is frequently and simultaneously framed as (*1*) a strength of the One Health approach in the process of understanding and solving complex problems associated with health challenges such as pathogen spillovers and pandemics and (*2*) a challenge regarding consensus on essential functions of One Health and the sets of knowledge, skills, and perspectives unique to a workforce adopting this approach. Progress in developing competency-based training in One Health has revealed coverage of various topics across fundamental, technical, functional, and integrative domains. Ensuring that employers value the unique characteristics of personnel trained in One Health will likely require demonstration of its usefulness, accreditation, and continuing professional development. These needs led to the conceptual framework of a One Health Workforce Academy (OHWA) for use as a platform to deliver competency-based training and assessment for an accreditable credential in One Health and opportunities for continuing professional development.

**Methods:**

To gather information about the desirability of an OHWA, we conducted a survey of One Health stakeholders. The IRB-approved research protocol used an online tool to collect individual responses to the survey questions. Potential respondents were recruited from partners of One Health University Networks in Africa and Southeast Asia and international respondents outside of these networks. Survey questions collected demographic information, measured existing or projected demand and the relative importance of One Health competencies, and determined the potential benefits and barriers of earning a credential. Respondents were not compensated for participation.

**Results:**

Respondents (*N* = 231) from 24 countries reported differences in their perspectives on the relative importance of competency domains of the One Health approach. More than 90% of the respondents would seek to acquire a competency-based certificate in One Health, and 60% of respondents expected that earning such a credential would be rewarded by employers. Among potential barriers, time and funding were the most cited.

**Conclusion:**

This study showed strong support from potential stakeholders for a OHWA that hosts competency-based training with opportunities for certification and continuing professional development.

## Background

The One Health approach arose from a growing realization that many human pathogens emerge from animal populations, while environmental systems mediate the potentiality and severity of spillover from one species to another, including amplification and spread resulting in pandemics [1–3]. However, the lessons learned over the past two decades regarding One Health and implementation of its strongest recommendations were insufficient to prevent a pandemic of the magnitude experienced with COVID-19. One of the lessons of the ongoing COVID-19 pandemic is the paucity of workers able to integrate and synthesize knowledge and competencies at the intersection of human health, animal health, and environmental ecosystems [4]. Ongoing evaluations show gaps in the preparation and training of the workforce needed to implement the One Health approach, particularly in under-resourced countries and regions [5]. Therefore, boosting the One Health workforce seems a good place to begin addressing human resources gaps in One Health competencies. These workforce gaps are recognized in simultaneous efforts occurring at the international level. For example, in 2022, the World Health Organization’s (WHO) Health Workforce Office launched a roadmap for building the public health and emergency workforce, including the development of essential functions of public health, as a foundation for competency-based training and continuing education [5]. Similarly, in 2022, the WHO, World Organizations for Animal Health (WOAH), and the Food and Agriculture Organization (FAO) of the United Nations (UN) launched an initiative to recast field epidemiology training programs in light of the One Health approach by defining sets of competency domains and guidelines for curriculum design, continuing professional education, and credentialing [6]. The FAO’s field training program (FTP) for wildlife, ecosystems, biodiversity, and the environment (FTP-WEBE) also developed a curriculum framework using a One Health approach [7]. These parallel efforts complement the initiative for a One Health Workforce Academy (OHWA) and facilitate cross-fertilization of ideas through collaborative participation and codevelopment of the One Health competency framework.

The current One Health workforce consists primarily of individuals who self-identify as working within the One Health framework at the intersection of human health, animal health, and environmental or ecosystem health [8]. In most cases, workforce personnel work within their discipline or profession in a particular sector while acknowledging the relevance of consulting or collaborating with individuals in other sectors. One Health practitioners thus have a wide range of backgrounds and expertise with the expectation that they will collaborate to implement programs to secure global health in the face of threats at the intersection of human and animal populations and ecosystems. Despite the increasing theoretical support for the One Health approach, gaps remain in matters of praxis and operationalization [9, 10]. Specifically, consensus remains elusive around the unique set of knowledge, skills, and essential functions associated with a One Health workforce and how often training and cultivation of such an approach should be updated [11–14]. The proposed OHWA could address gaps by providing training resources that are competency based, high quality, appropriate for international audiences, comprehensive, and truly multidisciplinary in nature [15, 16].

Employees who are certified in One Health are expected to work collaboratively to prevent pathogen spillover events, detect disease outbreaks, and respond to pandemics and other threats to global health security using the One Health approach [17]. While specific, in-depth, technical training is needed within individual sectors and disciplines, a credential in One Health could provide a bridge across specializations when addressing complex challenges, particularly for the development of functional skills such as collaboration and communication. Effective implementation of the One Health approach also requires that potential employers value and reward training in One Health based on the superior performance of credentialed personnel situated in positions that demand cross-sector understanding and development of sustainable solutions using this approach. A One Health credential should be valuable to employers as it is a comprehensive international resource for personnel interested in applying a One Health approach to solve complex problems, which is a sparsely occupied niche. The OHWA concept was proposed as a product of the consortium of partners under the United States Agency for International Development (USAID)-funded One Health Workforce-Next Generation (OHW-NG) project to fulfill the need for supplementary and complementary training at institutions offering academic and professional degree programs in disciplines within the One Health framework and to serve as a bridge among functional, technical, and experiential knowledge, skills, and competencies. A robust OHWA could fill the gaps among the various types of training at universities, which differ in terms of prioritized sets of competency domains, curriculum designs, modes of education delivery, and support for continuing professional development. As originally envisioned, the OHWA’s publicly accessible portal (https://onehealthworkforceacademies.org) is designed to provide international access to high-quality, readily available training materials and resources to engage One Health trainees at all levels, to support a community of practice, and to provide a repository of best practices for faculty members teaching the One Health approach. The future utility of this virtual academy platform is expected to be informed by the results of the current study. The OHWA was designed with international visibility in mind, offering information delivered through live, interactive, web content and a collection of unique resources in terms of quality and orientation toward career advancement. Given the mission of the OHWA to promote the development, delivery, institutionalization, accreditation, and employer recognition of training and educational activities in alignment with One Health knowledge, competencies and perspectives, the specific objectives include the following:

1. Supporting international and regional institution networks invested in training, employing, and empowering competent and credentialed One Health workforce personnel.
2. Supporting international and regional networks of faculty members and scholars to improve in their ability to fulfill their responsibilities in research, pedagogy, and practice of One Health.
3. Supporting communities of practitioners across academic disciplines and professions aligned with One Health.
4. Articulating international and regional career pathways through accredited credentials, attestation, and continuing profession development for in-service One Health employees.
5. Supporting employers seeking opportunities to engage trained preservice students and in-service employees in One Health.
6. Partnering with international agencies (e.g., the WHO Academy) [18] to leverage resources and share best practices related to the One Health workforce.

The purpose of this study was to investigate stakeholder needs and expectations for the OHWA and to align the objectives of the academy with the priorities of trainees, faculty, employers, and One Health practitioners. The results of the survey will directly inform further development of the OHWA. The study was designed to characterize potential barriers and benefits associated with an online academy for delivering training, assessing competence, and attesting qualifications for entering and prospering in the One Health workforce. We also sought to understand the demand for One Health training and the relative importance of various competencies in the educational configuration of One Health.

## Methods

### The One Health Workforce-Next Generation Network

The study was implemented through the One Health Workforce Consortium, which consists of the Africa One Health University Network (AFROHUN) [19], the Southeast Asian One Health University Network (SEAOHUN) [20] and the Global Partners’ team. These collaborative networks perform activities funded by USAID’s OHW-NG Project. The AFROHUN member countries include Cameroon, Cote D’ Ivoire, the Democratic Republic of Congo (DRC), Ethiopia, Kenya, Rwanda, Senegal, Tanzania, and Uganda. As of 2023, within these AFROHUN member countries, there are 18 member universities, with 26 member institutions. The SEAOHUN member countries include Cambodia, Indonesia, Lao, Malaysia, Myanmar, the Philippines, Thailand, and Vietnam. As of March 2023, within these member countries, there are 99 member universities and 182 member faculties. Collectively, the two networks include 110 universities, each offering or developing competency-based training and faculty development activities related to One Health. The Global Team is a group of partners engaged in the OHW-NG project that includes the University of California, Davis; Columbia University; Ata Health Strategies; EcoHealth Alliance; the University of California, Berkeley; the University of California, Irvine; the University of New Mexico; Labyrinth Global Health; the American Society for Microbiology; and the Smithsonian Institution, all of which are institutions based in the United States.

### Research protocol and the survey instrument

The research protocol and questions to be included in the stakeholder survey were drafted by a small group of global researchers funded by USAID to implement specific aims under the training and empowerment objective of the OHW-NG project [21]. The draft protocol and questions were shared with key active members of the AFROHUN and SEAOHUN to obtain feedback. The questionnaire instrument was piloted (*N* = 97) from September 2020 to December 2020. The survey was subsequently further refined, and the final draft of the protocol and questionnaire was submitted to the Institutional Review Board (IRB) of the University of California, Davis, for further review; this protocol was exempt from the need for additional review beyond the initial IRB approval. The IRB also reviewed and approved the human participant recruitment flier and the conduction of the survey in an online platform to ensure that the research was conducted according to international policy guidelines required for collecting information in a nonidentifiable way. The study protocol consisted of a single set of questions to be answered by participants. The IRB-approved research protocol used the online tool SurveyMonkey (https://www.surveymonkey.com/r/OHWASurvey). Information about consent to participate in the research was provided on the first webpage; consent was required before a respondent could access the questionnaire. Those who did not provide consent were excluded from the survey. In the pilot phase, we estimated that the survey questions could be answered within 15 min. The survey platform allowed language translation, which was provided upon request. This functionality was used to create French, Vietnamese, and Thai versions of the survey, which were generated using gold-standard translation practices, including translation and back-translation. Responses to the formal survey were collected from May 2021 to November 2021.

In soliciting participants from AFROHUN and SEAOHUN countries, we emphasized the need to include potential employers of graduates of the existing One Health training programs. Additionally, active key members of the AFROHUN and SEAOHUN were asked to share the survey with diverse members of their extended networks, with special consideration to sharing across multiple relevant sectors, including private, public, and education sectors as well as nongovernmental organizations (NGOs). While network participants were not excluded from taking the survey, a framework was provided to each key network member to generate diversity in respondents, including requesting dissemination of the survey outside of the academic sector. In addition to AFROHUN and SEAOHUN participants, we invited participants from institutions of higher learning that offer academic and professional degrees in the health sciences and have already integrated the concepts of One Health into their curriculum or who are planning to do so (and thus would likely generate alumni stakeholders of the OHWA). Through an internet search, we identified several institutions that have implemented certificates, diplomas, and master’s and doctorate curricula in One Health. For example, the University of Edinburgh in Scotland offers a Master of Science in One Health [22], and the University of Washington in Seattle offers a Master of Public Health in One Health [23]. The questionnaire was designed to ensure that we collected information from potential stakeholders on areas of agreement and diversity of opinions, including gaps and opportunities in the design of the structure and functions of the OHWA, with the aim of positioning it as an international asset for empowering the One Health workforce. The survey was implemented largely using a snowball technique, asking key members of the SEAOHUN and AFROHUN to share it broadly across their professional networks. Members were also encouraged to share the survey across sectors (e.g., education sector, governmental sector, and NGOs) to ensure diversity among respondents. Members from each country network within the regional networks were also targeted to ensure that responses were collected from as many countries as possible. The data were collected, managed and stored through Survey Monkey, a secure online survey platform. The survey results were analyzed with descriptive statistical methods in Microsoft Excel, including the calculation of weighted averages. Figures were also generated using this software.

## Results

**Fig. 1:**
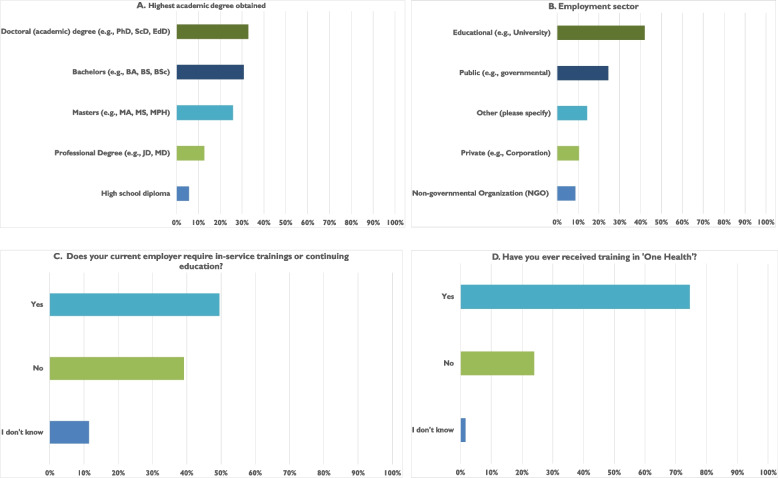
Background information about survey participants’ academic qualifications, training, and employment

The survey was completed by 231 participants from 24 countries. Their country of employment and affiliation with One Health University Networks (OHUNs) are reported in Table 1. The highest number of participants in each of the three regions were from the following countries: Kenya (in the Africa region), Malaysia (in the Southeast Asia region), and the United States (representing other international regions). Most of the respondents had earned advanced degrees (> 45% had doctorate degrees) and most were affiliated with academic institutions (> 55%). Most respondents (> 75%) had been trained in One Health, although the depth of their training was not assessed, and > 45% noted that their employer expected a form of continuing education and professional development (Fig. 1).

**Table 1:** Survey participants’ national origin and affiliation with a specific network of universities committed to developing and implementing the One Health approach in educational programs

One Health geographic network	Country	Number of participants
Africa One Health University Network	Kenya	19
	Uganda	12
	Ethiopia	7
	Cameroon	4
	DRC	4
	Rwanda	4
	Senegal	4
	Tanzania	4
	Cote D’Ivoire	1
Southeast Asia One Health University Network	Malaysia	71
	Thailand	35
	Vietnam	31
	Indonesia	18
	Cambodia	2
	Philippines	1
Global	United States	4
	Nigeria	3
	Algeria	1
	Bangladesh	1
	Benin	1
	Guinea	1
	Mauritania	1
	South Korea	1
	United Kingdom	1
Total		231

**Fig. 2:**
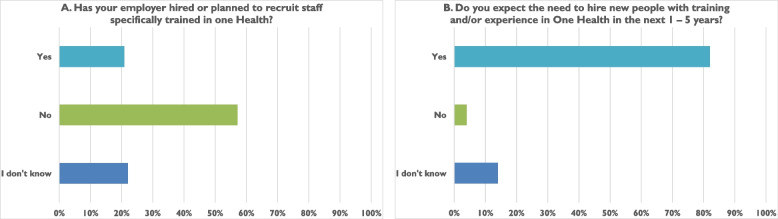
Respondents’ knowledge of current and prospective employment opportunities specifically requiring training in One Health

Employment opportunities advertised specifically for those trained in One Health are currently rare, consistent with respondent reports; fewer than 20% of participants noted that their current place of employment had hired or planned to hire individuals specifically trained in One Health. However, when we asked participants to predict the situation 5 years in the future, more than 80% indicated that their employers would likely recruit One Health-trained personnel (Fig. 2).

**Fig. 3:**
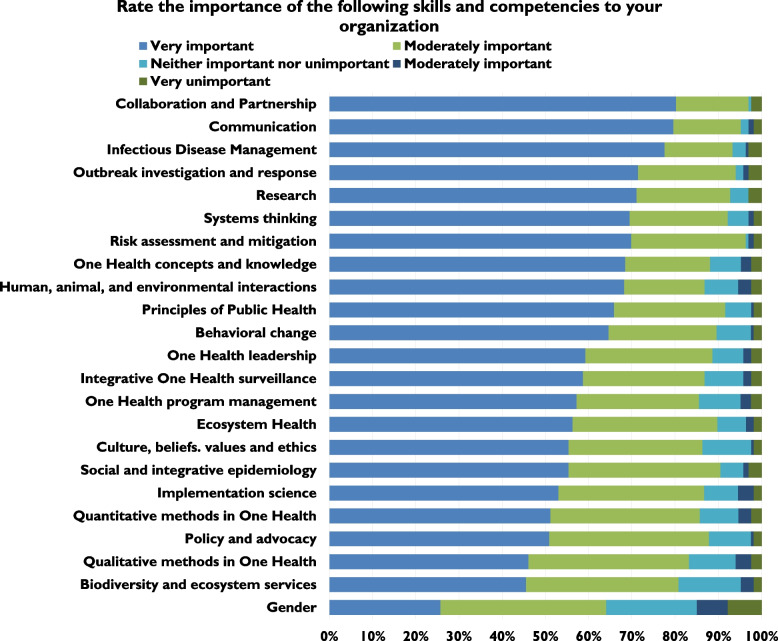
Survey respondents’ assessment of the relative importance of One Health competencies in terms of the current functions of the organization at which they are employed

Several questions in the survey focused on quantifying the importance of key competencies and skills related to the One Health approach [24]. Participants were asked to rate 23 skills in terms of importance (from ‘very important’ to ‘very unimportant’) to the current functions of their employer. We also provided the opportunity to nominate competencies or subjects not included in the list. Twenty-two of the 23 listed competencies were rated as ‘very important’ by at least 50% of respondents who answered that question. The only competency that did not meet this threshold was gender. All 23 competencies were ranked as ‘very important’ or ‘moderately important’ by at least 80% of the respondents (Fig. 3). This shows a high level of agreement across the respondents that the 23 listed skills and competencies are important to their employers and institutions.

Analysis of the ranked competencies using a weighted average revealed that those ranked as the highest importance across all participants were (in descending importance) ‘collaboration and partnership’, ‘communication’, and ‘infectious disease management’ (Table 2). Those with the four lowest ranking scores were ‘qualitative methods in One Health’, 'biodiversity and ecosystem services’, and ‘gender’. To clarify the core competencies most important to employers, the results of these rankings were separated by sector of employment, including the public/governmental sector, education sector, private sector, and NGOs. Gender was ranked as least important (23/23) across all sectors except by those in NGOs, where it was tied for fourth-least important (19/23).

**Table 2:**
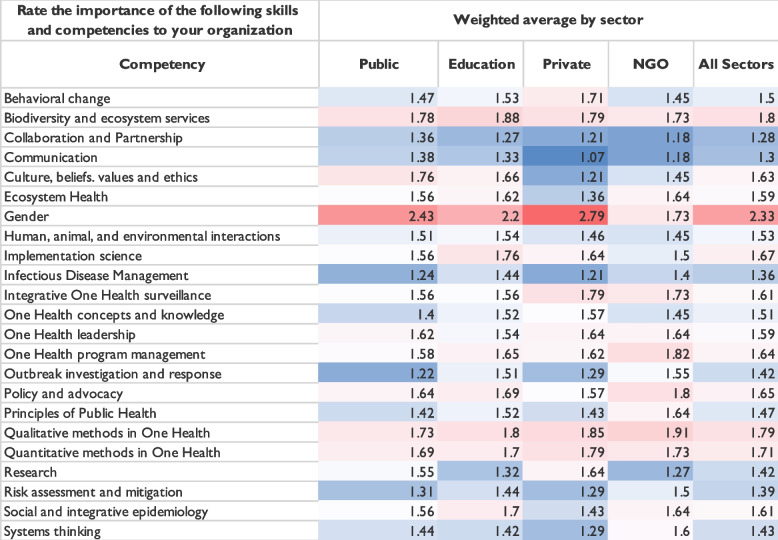
Weighted average rankings of the importance of competencies to the respondents’ organization by sector

Regarding the weighted average of skills and competencies, ‘collaboration and partnership’ and ‘communication’ were ranked in the top three skills across all sectors, with the exception of the public/governmental sector. Those in this sector selected the following three skills as the most important: ‘outbreak investigation’, ‘infectious disease management’, and ‘risk assessment and mitigation’. ‘Collaboration and partnership’ and ‘communication’ were 4^th^ and 5^th^ most important skills, respectively. This might indicate that those in the public sector value ‘technical’ skills over ‘functional’ skills. Similarly, those in the public sector were the only group in which ‘qualitative methods in One Health’ were not listed in the bottom three. There were only a few significant differences in sector rankings of the competencies according to a 95% confidence interval (*p* = 0.05). For example, regarding the importance of ‘research’, 59.09% of those in the public/governmental sector ranked it as ‘very important’, whereas 82.05% of those in the education sector ranked it as ‘very important’. However, the weighted averages were not entirely dissimilar, as many governmental employees (31.82%) rated ‘research’ as ‘moderately important’.

**Fig. 4:**
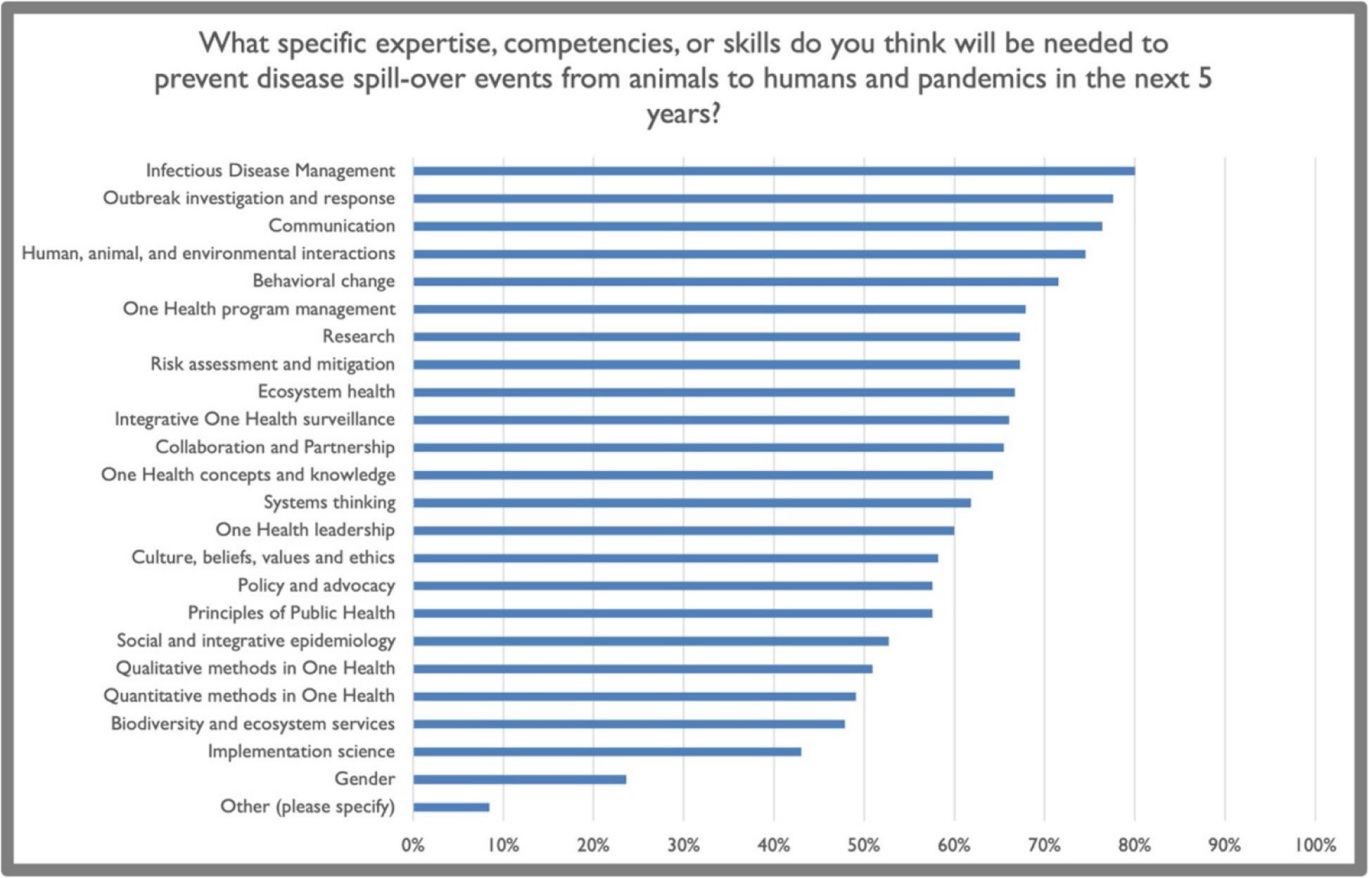
Survey participants’ assessment of the relevance of current One Health competencies to disease spillovers and pandemic prevention. Note that the survey was conducted during the COVID-19 pandemic

Figure 4 displays answers to the question regarding the relative importance of training in the One Health competencies for preventing pathogen spillover events and pandemics in the future. The results were similar to those shown in Fig. 3, with ‘gender’ rated as the least important. ‘Policy and advocacy' also received a low rating. In contrast, functional competencies such as ‘systems thinking’, ‘collaboration and partnerships’, and ‘behavior change’ were rated highly. A write-in answer repeated by multiple participants was ‘antimicrobial resistance’, which underscores the importance of defining the integration of skills and competencies necessary to address specific emerging issues or complex challenges, including the intersection of climate change and the One Health approach.

**Fig. 5:**
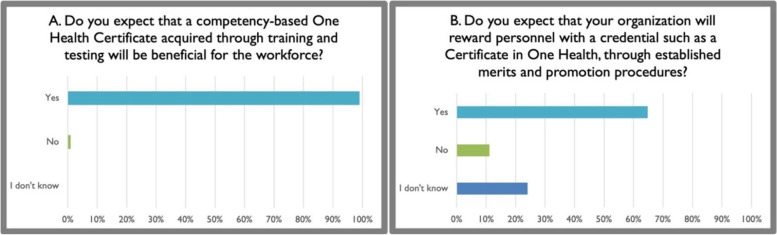
Survey participants strongly expected benefits from a competency-based credential in One Health (**A**) and were more reserved regarding the impact on advancement and promotion (**B**)

**Fig. 6:**
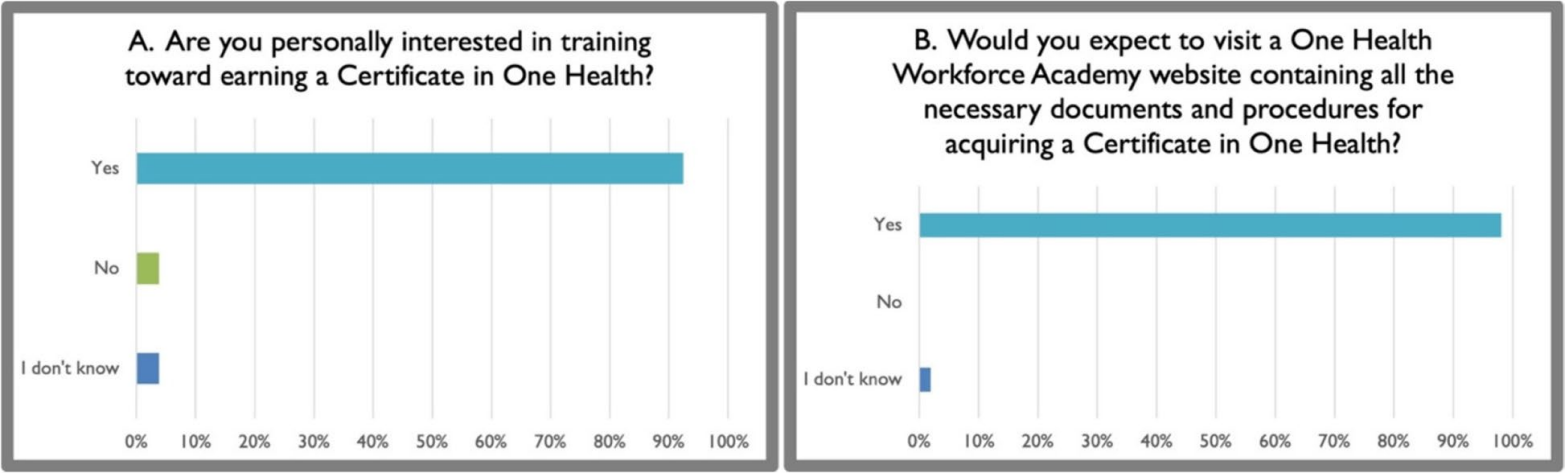
Survey participants’ interest in earning a competency-based certificate in One Health (**A**) and prospective role of the One Health Workforce Academy in delivering the credential (**B**)

**Fig. 7:**
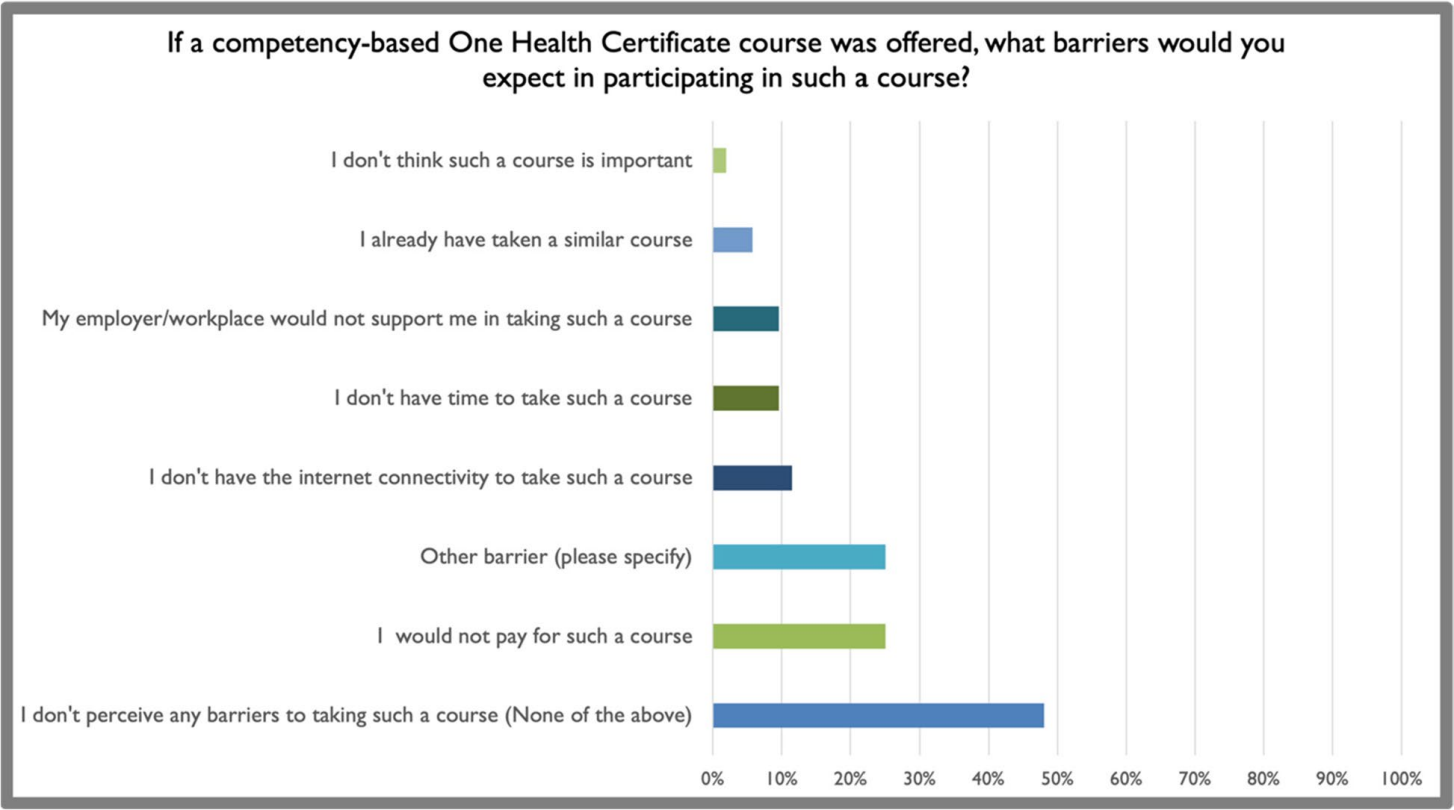
Survey respondents’ perceived barriers to enrolling in a competency-based course to earn a certified credential in One Health

More than 90% of participants expected that a competency-based credential such as a One Health certificate earned after training and assessment would be beneficial for the workforce. However, only approximately 60% expected that current employers and supervisors would reward such a credential with promotion, merits, and/or placement in positions of decision-making regarding One Health-oriented programs (Fig. 5). When examined by sector, 67.95% of respondents in the education sector reported that they expected that their organization would reward personnel for such a credential, whereas only 60% of those in the public sector, 42.86% of those in the private sector and 40% of those in NGOs reported that they had the same positive expectation of a reward. This result highlights a potential gap in reward pathways for those working in NGOs who might be interested in receiving a One Health credential. Nevertheless, more than 90% of respondents noted that they were personally interested in earning a certificate in One Health, and approximately the same proportion of respondents expected to visit the OHWA website to enroll in training toward certification when the curriculum becomes available (Fig. 6). Approximately 50% of respondents perceived no barriers in pursuing online-based training toward a certificate in One Health through the OHWA. Those who perceived one or more barriers noted limitations in funding, time, and reliable access to internet services (Fig. 7). Perceived gaps did not differ significantly among sector of employment.

To obtain further fine-grained understanding of the survey responses, particularly those from AFROHUN and SEAOHUN member countries, the responses were examined by country of employment. There were no significant differences in responses. This can be attributed to the skewedness of the sample to a few countries, which will be discussed in the limitation section.

## Discussion

The survey revealed broad support for a OHWA to host competency-based training toward a credential and for continuing education and professional development. The survey revealed a diverse stakeholder landscape across sectors with diverse requirements and needs for continuing professional development in the use of the One Health approach. Despite this diversity, the results indicated strong interest among individuals in earning a credential and continuing professional development in One Health. The survey also indicated resounding understanding of the importance of developing such a workforce both for organizations and prevention of future pandemics.

However, the survey indicated a gap in current organizational support to complement and bolster the interest of individuals. Despite expectations for both the need to hire those trained in One Health and that such training will benefit the workforce, employers are not specifically attempting to recruit individuals with One Health skills. Despite the widespread understanding that One Health training is beneficial to the workforce, most relevant employers are not currently requiring One Health-specific training for continuing professional development or other on-the-job training requirements. Additionally, the survey suggested that employees do not expect to be rewarded for such One Health training and that there is a diverse perception of necessary skills to address One Health-based challenges to global health security. These issues highlight the need to sensitize employers to the One Health approach and for increased visibility of the competency-based approach to One Health training.

The survey highlighted respondents’ perceptions of the importance of a collection of One Health skills and competencies to their employer. A critical result of the survey was that the competency ‘gender’ was described as the least important to the employers of respondents and had the highest numeric weighted average. We examined this low ranking by sector and found that those working in NGOs did not rank gender in the bottom three competencies. However, all other sectors did. While the survey did not collect further qualitative data regarding the explanations for this difference, the results emphasize not the absolute importance of competency per se but rather the need to build awareness of gender as a critical One Health skill at the institutional/organizational level moving forward.

Surprisingly, the competency ‘biodiversity and ecosystem services’ was ranked relatively low regarding its perceived importance to employers as well as its perceived importance (by respondents) in preventing pandemics. In terms of importance to employers, this competency was ranked second lowest (22/23) using the weighted average. In addition, ‘biodiversity and ecosystem services’ was ranked third-lowest regarding importance in preventing pandemics, behind only gender and implementation science. Historically, environmental and ecosystem contexts have been a neglected aspect of the One Health paradigm [25, 26]. This may explain the lack of perceived importance of these disciplines for employers of One Health experts. Similar to gender, this is not an indication of the absolute importance of ‘biodiversity and ecosystem services’ as a One Health competency but rather of the need to communicate applications, linkages and urgency to understand and apply these skills.

The survey results highlight practical aspects of workforce development and training for further investigation, including articulation of goals toward accredited competency-based training in One Health. The majority of respondents were interested in obtaining a certificate in One Health as well as accessing training materials through an online platform. However, the barriers to using or accessing such materials included time, which underscores a reoccurring challenge of training in-service professionals. Another common barrier was willingness or ability to pay. This challenge highlights the importance of considering funding during the design of future credentialed training programs, with consideration for sustainability and equity in the delivery of training programs. In many cases, respondents reported already being required to attend training in continuing professional development and other on-the-job training. Given that time and costs were identified as barriers, it will be important to design training programs that complement or are synergetic with existing employer requirements. These results will also be useful for parallel efforts at the international level, including the WHO-WOAH-FAO-UNEP partnership, which is developing competency-based field epidemiology training programs based on the One Health approach.

### Strengths and limitations

The survey used a snowball sampling method. This sampling method was chosen to emphasize the qualitative approach of this study to examining particular types of employees working within the One Health context. The participant sample was nonrandom because we actively encouraged key network participants to share the opportunity to participate among their respective networks. This method was chosen to ensure that the survey was disseminated to those working in One Health outside of our own network. In addition, those disseminating the survey were encouraged to share the survey broadly outside of educational institutions to obtain representation from other groups, including employers.

The survey reached many countries and included representation from diverse sectors; however, some countries and sectors were represented more strongly than others. It is unclear why Kenya and Malaysia had high response rates, but we suspect that there was more vigorous sharing of the opportunity to participate in the research from the university partners in these countries, resulting from our snowball approach. Additionally, the high level of engagement from those in the education sector was not surprising, given that the AFROHUN and SEAOHUN are composed of universities.

The survey was likely disseminated only to participants already familiar with One Health. Based on our survey aims, we do not consider this a source of bias because the purpose of the survey was to investigate potential stakeholders of the OHWA. However, the next step is to investigate the perceptions and interest of more distant parties, such as those largely unaware of the One Health approach but working in public health, ecology, social sciences, or other related professions and disciplines. A specific source of bias may be associated with use of the AFROHUN and SEAOHUN and their preconceived expectations of earning a One Health credential. Almost all network participants indicated interest in earning credentials through the OHWA. This group can be considered our closest stakeholders and represents only the first level of outreach for the development of a globally recognized training platform.

## Conclusion

The training and empowerment of personnel needed to secure global health requires interdisciplinary and interprofessional understanding and collaboration [27]. A One Health approach fills the wide gaps between well-established and relatively independent sectors in public and private health agencies and academic institutions. Well-defined competencies and transparent career paths universally valued by employers are needed for the One Health workforce to deliver essential functions [28]. This study demonstrates unequivocally strong support by stakeholders, including employers, for a OHWA that hosts competency-based training with opportunities for certification and continuing professional development.

## Data Availability

All data were collected and compiled through the published questionnaire based on an online survey tool (https://www.surveymonkey.com/r/OHWASurvey).

## Abbreviations

AFROHUN: Africa One Health University Network
CPD: Continuing professional education and development
OHWA: One Health Workforce Academy
SEAOHUN: Southeast Asia One Health University Network
